# Polymer formulated self-amplifying RNA vaccine is partially protective against influenza virus infection in ferrets

**DOI:** 10.1093/oxfimm/iqac004

**Published:** 2022-06-27

**Authors:** P F McKay, J Zhou, R Frise, A K Blakney, C R Bouton, Z Wang, K Hu, K Samnuan, J C Brown, R Kugathasan, J Yeow, M M Stevens, W S Barclay, J S Tregoning, R J Shattock

**Affiliations:** Department of Infectious Disease, Imperial College London, London W2 1PG, UK; Department of Infectious Disease, Imperial College London, London W2 1PG, UK; Department of Infectious Disease, Imperial College London, London W2 1PG, UK; Department of Infectious Disease, Imperial College London, London W2 1PG, UK; Department of Infectious Disease, Imperial College London, London W2 1PG, UK; Department of Infectious Disease, Imperial College London, London W2 1PG, UK; Department of Infectious Disease, Imperial College London, London W2 1PG, UK; Department of Infectious Disease, Imperial College London, London W2 1PG, UK; Department of Infectious Disease, Imperial College London, London W2 1PG, UK; Department of Infectious Disease, Imperial College London, London W2 1PG, UK; Departments of Materials and Bioengineering, Institute of Biomedical Engineering, Imperial College London, London SW7 2AZ, UK; Departments of Materials and Bioengineering, Institute of Biomedical Engineering, Imperial College London, London SW7 2AZ, UK; Department of Infectious Disease, Imperial College London, London W2 1PG, UK; Department of Infectious Disease, Imperial College London, London W2 1PG, UK; Department of Infectious Disease, Imperial College London, London W2 1PG, UK

**Keywords:** RNA vaccine, influenza, ferret

## Abstract

COVID-19 has demonstrated the power of RNA vaccines as part of a pandemic response toolkit. Another virus with pandemic potential is influenza. Further development of RNA vaccines in advance of a future influenza pandemic will save time and lives. As RNA vaccines require formulation to enter cells and induce antigen expression, the aim of this study was to investigate the impact of a recently developed bioreducible cationic polymer, pABOL for the delivery of a self-amplifying RNA (saRNA) vaccine for seasonal influenza virus in mice and ferrets. Mice and ferrets were immunized with pABOL formulated saRNA vaccines expressing either haemagglutinin (HA) from H1N1 or H3N2 influenza virus in a prime boost regime. Antibody responses, both binding and functional were measured in serum after immunization. Animals were then challenged with a matched influenza virus either directly by intranasal inoculation or in a contact transmission model. While highly immunogenic in mice, pABOL-formulated saRNA led to variable responses in ferrets. Animals that responded to the vaccine with higher levels of influenza virus-specific neutralizing antibodies were more protected against influenza virus infection. pABOL-formulated saRNA is immunogenic in ferrets, but further optimization of RNA vaccine formulation and constructs is required to increase the quality and quantity of the antibody response to the vaccine.

## INTRODUCTION

RNA vaccines have been a key part of the response to the COVID-19 pandemic [1]. However, they are still a relatively new technology and further optimization is important for future vaccines, both against endemic and pandemic pathogens. One approach that may have benefits in dose reduction is the use of self-amplifying RNA (saRNA) vaccines. These vaccines are based on alphaviruses that in addition to expressing a target antigen also contain non-structural genes encoding replication machinery, which means they can make copies of themselves within the transfected cell [2]. We have previously observed in mice that saRNA vaccines can protect against influenza virus infection with at least 64-fold less material than non-replicating mRNA vaccines [3]. However, in a first-in-human clinical study using a saRNA vaccine expressing the SARS-CoV-2 spike protein, not all volunteers produced an antibody response to the vaccine, with 61% seroconverting in the 10 µg group [4]. Understanding why the vaccine was not universally immunogenic is important in the ongoing development of this promising platform.

One consideration that may be important for improving immunogenicity is altering the formulation in which the saRNA is delivered. The first-in-human saRNA trial used a lipid nanoparticle (LNP) formulation, but other formulation approaches have been investigated in pre-clinical models, including polymers [5]. We have recently developed a bioreducible, linear, cationic polymer called pABOL [6], which was immunogenic in mice when formulated with saRNA. We wanted to investigate whether the same formulation was effective in other species and for a range of antigens. Influenza virus is an important respiratory pathogen, with the potential to cause pandemics [7]. It also has a considerable endemic burden, contributing to 294 000–518 000 deaths globally in a normal year [8]. The development of RNA vaccines for influenza virus may be an important control measure, due to their speed of deployment (for pandemic viruses) and the need for adaptation associated with vaccines grown in eggs (for seasonal viruses).

Ferrets are a well-established model of influenza virus infection and transmission since both human and avian isolates replicate in the ferret airway leading to clinical signs similar to those seen in infected humans [9]. A small number of RNA vaccine studies have been performed in ferrets, three using mRNA [10–12] and one using saRNA [13]. These studies utilized lipid-based formulations, either LNP [10, 12] or a cationic nano-emulsion [13]; in one study [11], it was not explicitly stated what was used, though the study cites an earlier publication employing a liposome/protamine formulation [14]. The impact of cationic polymers on RNA immunogenicity has not been investigated in the ferret model.

In the current study, we investigated the immunogenicity of pABOL-formulated saRNA for a vaccine against influenza virus in mice and ferrets. We explored the relationship between induced anti-viral antibody and protection against influenza virus infection in both a direct infection model and a transmission model. We observed that pABOL induces a variable level of protection in ferrets, indicating that further optimization is required for pABOL-formulated saRNA vaccines in this model.

## MATERIALS AND METHODS

### saRNA construct

saRNA was synthesized from a backbone plasmid vector based on a Trinidad donkey Venezuelan equine encephalitis strain (VEEV) alphavirus genome as previously described [15]. The genes of interest (GOI) for *in vivo* immunogenicity studies were haemagglutinin (HA) from the H1N1 A/California/07/2009 strain and HA from the H3N2 Japan (A/Japan/WRAIR1059P/2008(H3N2).

### *In vitro* transcription of saRNA

Briefly, uncapped RNA was prepared using 1 μg of linearized DNA template in a MEGAScript reaction (Ambion, UK) according to the manufacturer’s protocol. Transcripts were then purified by overnight LiCl precipitation at −20°C, pelleted by centrifugation at 14 000 RPM for 20 min at 4°C, washed once with 70% ethanol, centrifuged at 14 000 RPM for 5 min at 4°C and then resuspended in UltraPure H_2_O (Ambion). Purified transcripts were then capped using the ScriptCap m7G capping system (CellScript, Madison, WI, USA) and ScriptCap 2′-0-methyltransferase kit (CellScript) simultaneously according to the manufacturer’s protocol. Capped transcripts were then purified by LiCl precipitation, as detailed above, resuspended in UltraPure H_2_O and stored at −80°C until formulation.

### Vaccine formulation

pABOL (*M*_w_ = 8 kDa) was synthesized using a previously reported protocol [16].

### Mice

The 6–8-week-old female BALB/c mice were obtained from Charles River UK Ltd (Rugby, UK) and kept in specific-pathogen-free (SPF) conditions in accordance United Kingdom’s Home Office guidelines. All work was approved by the Animal Welfare and Ethical Review Board (AWERB) at Imperial College London. Mice were immunized with 1 µg of saRNA in a 100-µL volume intramuscularly in a prime boost regime at 0 and 4 weeks. Six weeks after first dose, mice were infected intranasally with 3 × 10^4^ Plaque Forming Units (PFU) H1N1 Cal/09 in 100 µL volume; where used mice were anaesthetized with inhaled isoflurane. Seven days after infection, mice were culled; and blood and lung tissue collected. Antibodies were assessed by semi-quantitative ELISA as previously described [17]. Cells were recovered from homogenized lungs and counted. Analysis of influenza virus-specific CD8 cells was performed by flow cytometry as described [18]. Live cells were suspended in Fc block (Anti-CD16/32, BD) in Phosphate Buffered Saline (PBS)–1% Bovine Serum Albumin (BSA) and stained with surface antibodies: influenza virus A H1 HA_533-541_ IYSTVASSL Pentamer R-PE (Proimmune, Oxford, UK), CD3-FITC (BD, Oxford UK), CD4-APC (BD) and CD8-APC Alexa750 (Invitrogen, UK).

### Ferrets

Experiments were performed in a containment level 2 laboratory. Outbred female ferrets (28–32 weeks old) weighing 750–1000 g were used (Highgate Farms). Ferrets were confirmed to be seronegative for influenza virus A virus NP protein by competitive ELISA (ID.vet) [19]. For virus inoculation, ferrets were lightly anesthetized with ketamine (22 mg/kg) and xylazine (0.9 mg/kg).

#### Direct infection study (H1N1)

Ferrets were immunized with 5, 20 or 80 µg of saRNA expressing H1 in a 50-µL volume in the hindleg muscle at 0 and 4 weeks and challenged at 6 weeks after the first immunization. Animals were infected with 10^6^ PFU pandemic H1N1 virus (A/California/07/2009) in PBS (100 µL per nostril).

#### Contact transmission study

The effect of vaccine on direct contact transmission was assessed by employing an experimental setup cohousing naïve donor and immunized sentinel ferrets [20]. All 12 direct contact sentinel ferrets were pre-exposed to 10^6^ PFU pandemic H1N1 virus (A/California/07/2009) 4 weeks prior to immunization (week −4). Animals were then immunized with 20 µg saRNA expressing H3N2 or rabies virus glycoprotein as a control antigen in a 50-µL volume at 0 and 6 weeks and exposed to infected donor animals 12 weeks after the first immunization. Naïve donor ferrets were inoculated intranasally with 5 × 10^5^ PFU A/Japan/WRAIR1059P/2008(H3N2) diluted in PBS (100 μL per nostril). On day 1 post inoculation of donor, two immunized direct contact sentinels were co-housed with each donor animal.

#### Sampling

In both studies, all animals were nasal-washed daily, while conscious, by instilling 2 mL PBS into the nostrils, and the expectorate was collected in modified 250 mL centrifuge tubes. Ferrets were weighed daily post-infection and body temperature was measured daily via subcutaneous IPTT-300 transponder (Plexx B.V, Netherlands). Bedding litter in the cage was changed daily. At 14 days post infection (DPI), ferrets were injected with a non-reversible anaesthetic consisting of ketamine (≥25 mg/kg) and xylazine (≥5 mg/kg), before sacrifice by intracardiac injection of sodium pentobarbitone (≥1000 mg/kg), at which time blood was collected by cardiac puncture.

### Viral plaque assay

For viral plaque assays, Madin-Darby Canine Kidney (MDCK) cells and MDCK-SIAT cells were employed for H1N1 and H3N2, respectively. Cells were grown in Dulbecco’s modified Eagle’s medium (DMEM) supplemented with 10% (v/v) foetal bovine serum and 1% penicillin/streptomycin (Sigma-Aldrich). Briefly, fresh nasal wash samples were titrated on the day of collection and kept on ice till titration. MDCK cells were inoculated with 100 µL serially diluted samples and overlaid with 0.6% agarose (Oxoid) in supplemented DMEM (1× MEM, 0.21% BSA V, 1 mM l-Glutamate, 0.15% sodium bicarbonate, 10 mM HEPES, 1× penicillin/streptomycin, all Gibco and 0.01% Dextran DEAE, Sigma) with 2 µg trypsin (Worthington)/mL and incubated at 37°C for 3 days. The limit of virus detection in the plaque assays was 10 PFU/mL.

### Ferret antibody analysis

#### Antigen-specific ELISA

Briefly, 1 μg/mL of antigen-coated ELISA plates, coated O/N at 4°C in PBS, was blocked with 1% (w/v) BSA/0.05% (v/v) Tween-20 in PBS for 1 h at 37°C. After washing, diluted samples were added to the plates and incubated for 1 h, washed and 100 µL of a 1:2000 dilution of anti-ferret H + L IgG-HRP (Bethyl Laboratories Inc., USA) was added to each well. Standards were prepared by coating ELISA plate wells with Ferret IgG Purified Native protein (Antibody Research Corporation, USA), starting at 1000 ng/mL and titrating down with a 5-fold dilution series in PBS, O/N at 4°C and then blocking with PBS/1% (w/v) BSA/0.05% (v/v) Tween-20. Standard ELISA wells were incubated with the assay buffer during the experimental sera sample incubation. Samples and standards were developed using TMB (3,3′–5,5′-tetramethylbenzidine) and the reaction was stopped after 5 min with Stop solution (Insight Biotechnologies, UK). Absorbance was read on a spectrophotometer (VersaMax, Molecular Devices) with SoftMax Pro GxP v5 software.

#### Haemagglutinin inhibition assay

Briefly, sera was incubated with receptor-destroying enzyme (RDE II) (Denka Seiken Co.) at a ratio of 3 volumes of RDE to 1 volume sera and incubated at 37°C for 16 h and then heat inactivated at 56°C for 30 min. Then, 6 volumes of serum-free DMEM supplemented with 1 μg/mL TPCK-Trypsin (Thermo) were added to each sample and subsequently serially diluted 1:2 in PBS to a final volume of 25 μL and combined with 25 μL of working virus solution (4 HAU/25 μL). PBS was used as a negative control and virus was used as a positive control. The plates were incubated at RT for 30 min and then 50 μL of 0.5% red blood cells (Turkey Blood in Alsevers, ENVIGO) was added to each well and allowed to settle for 30 min at RT. The haemagglutination inhibition (HAI) titre was then recorded for each well, which was defined as the highest dilution that causes complete inhibition of haemagglutination.

#### Influenza virus microneutralization assay

Briefly, MDCK cells were seeded at 10 000 cells/well in cDMEM in a 96-well plate. Sera was prepared with RDE and TPCK-Trypsin and heat-inactivated as detailed in the HAI assay above and then diluted in 1:5 serial dilution in serum-free DMEM supplemented with penicillin/streptomycin, l-glutamine and 1 μg/mL TPCK-Trypsin. Samples were then diluted with an equal volume of virus at a concentration of 100 TCID50 in 50 μL and incubated for 1 h at 37°C and then added to MDCK cells and cultured for 24 h at 37°C. Cells were then fixed with cold 80% acetone and quantified using an influenza virus nucleoprotein ELISA. Plates were blocked with 5% non-fat milk in PBS + 0.05% Tween 20 for 1 h, then treated with rabbit anti-NP antibody (Thermo) diluted 1:1000 for 1 h and mouse anti-rabbit IgG-HRP (Santa Cruz) diluted 1:5000 for 1 h. Plates were developed using TMB solution for 5 min at RT before addition of stop solution and then read at OD450/OD800 to allow for the IC50 to be calculated for each sample.

### Statistical analysis

Calculations as described in figure legends were performed using Prism 9 (GraphPad Software Inc., La Jolla, CA, USA). Principal component analysis (PCA) was performed using the ‘stats’ package in R V 3.3.1 [21] and visualized using the R packages ‘ggfortify’ [22] and ‘scatterplot3d’ [23].

## RESULTS

### pABOL-formulated saRNA induces an equivalent antibody response to polyethylenimine in mice

**Figure 1:**
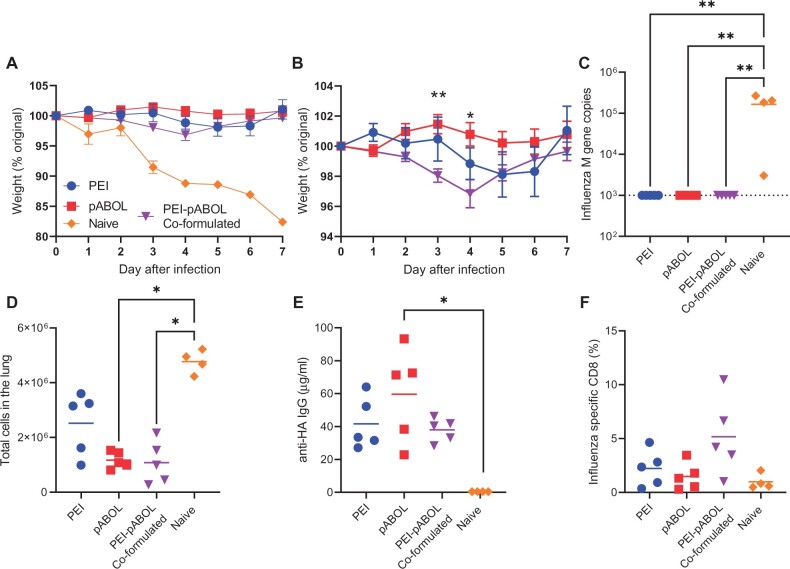
pABOL-formulated saRNA protects mice against influenza infection. Female BALB/c mice were intramuscularly immunized with saRNA encoding HA formulated with pABOL or PEI at 0 and 4 weeks. Mice were infected intranasally with influenza virus at 6 weeks and weight loss was measured after infection (**A**, **B**). Viral load (**C**), lung cell counts (**D**), anti-HA antibodies (**E**) and lung CD8 responses (**F**) on day 7 after infection. *N* = 5 mice per group, points represent individual animals (C–F) or means (A, B); Panel B *P < 0.05, **P < 0.01 between pABOL and PEI/pABOL group by two-way ANOVA. Panels C–E **P < 0.01, ***P < 0.001 by Kruskal–Wallis with post test.

pABOL-formulated RNA induces limited systemic inflammation, which might limit downstream immunogenicity, as some immune cell recruitment is required [16]. Another polymer frequently used to deliver nucleic acid vaccines, polyethylenimine (PEI) has been shown to be considerably more inflammatory [24]. We wanted to compare pABOL with PEI, with the hypothesis that pABOL might be safer and better tolerated due to its lower immunogenicity and inherent biodegradability under reducing conditions. We also wanted to explore the impact of combining PEI and pABOL as the PEI-induced inflammation might modulate the response. BALB/c mice were immunized intramuscularly with 1 µg saRNA expressing HA from Cal/09 H1N1 influenza virus formulated with either *in vivo* jetPEI, pABOL alone or *in vivo* jetPEI co-formulated with pABOL. Mice were immunized at 0 and 4 weeks in a prime boost regime and then challenged intranasally with influenza virus at 6 weeks. Weight was measured after infection (Fig. 1A). The previously naïve animals lost 20% body weight by day 7. All three formulations gave significant protection against weight loss compared with the naïve animals. Comparing the three formulations, mice immunized with saRNA in co-formulated PEI-pABOL lost a small amount of weight, which was significantly more than those mice immunized with pABOL alone on days 3 and 4 (Fig. 1B). There was no detectable viral RNA on day 7 after infection in any of the immunized groups (Fig. 1C). Vaccine naïve animals had significantly more cells in the lungs after infection (Fig. 1D), which we have previously observed correlates with airway inflammation [25]. To assess the adaptive immune response, we measured antibody and viral-specific CD8 cells after infection. Antibody responses were measured on day 7 after infection, the pABOL alone immunized group had significantly more antibody than the naïve group (Fig. 1E). There were detectable influenza virus-specific CD8 cells in all of the immunized groups, but no difference between the formulations (Fig. 1F). From this, we concluded that pABOL-formulated saRNA is equivalent to PEI and offers marginally better protection than PEI-pABOL co-formulation and therefore focused on pABOL for further studies.

### pABOL-formulated saRNA induces a variable antibody response in ferrets

Having observed that pABOL-formulated saRNA induces a protective immune response in mice, we wanted to investigate whether it was protective in larger animals. Ferrets are a well-established model of influenza virus infection and transmission. Six female ferrets were intramuscularly immunized with 5, 20 or 80 µg saRNA encoding HA formulated with pABOL in a prime boost regime at 0 and 4 weeks.

**Figure 2:**
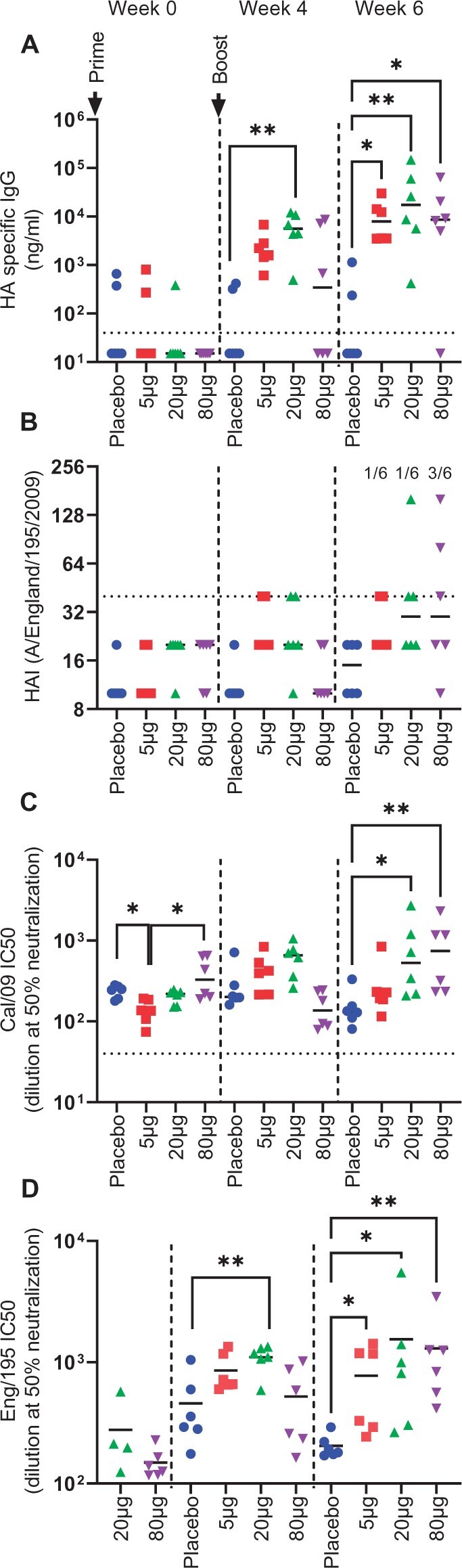
pABOL-formulated saRNA induces a variable antibody response in ferrets. Female ferrets were intramuscularly immunized with 5, 20 or 80 µg saRNA encoding HA formulated with pABOL at 0 and 4 weeks. Blood was collected to measure anti-HA antibody responses at 0, 4 and 6 weeks; responses were measured by ELISA (**A**), HAI (**B**) and microneutralization against Cal09 (**C**) or ENG/195 (**D**). *N* = 6 ferrets per group, points represent individual animals, Kruskal–Wallis test **P < 0.01, ***p < 0.001.

Serum was collected and tested for antibody responses. Antibody was assessed by ELISA binding assay (Fig. 2A). There was some low baseline reactivity to HA, in some of the animals, which reflects the outdoor rearing of the animals. After the prime, only the 20 µg group had a significantly higher antibody titre than the control animals, the 80 µg was lower than the 20 µg group, which may reflect a dose response relationship to the vaccine. After the boost, all immunized groups had a significantly greater antibody titre than the control animals. The antibody was then tested for function using a haemagglutination inhibition (HAI) assay. We used a titre of >1:40 to indicate seroconversion, as it is a commonly used measurement of protective seroconversion for influenza virus [26]. After two doses of vaccine, 50% of the animals in the high-dose group had seroconverted (Fig. 2B). As an alternative functional assay, we assessed the capacity to neutralize virus through a micro-neutralization assay against neutralization of the virus antigen in the construct (Cal/09: Fig. 2C) and a similar H1N1 strain (Eng/195: Fig. 2D). The neutralization titres reflected the ELISA binding titre. There was some baseline reactivity and the 5 µg group were significantly lower than the placebo and 80 µg groups for Cal/09 titre. After priming, only the 20-µg group had a significantly greater anti-ENG 195 neutralizing titre than the control animals (Fig. 2D). After the booster immunization, all vaccinated groups had a significantly greater neutralizing titre than control. Having seen that the pABOL-formulated vaccine was able to induce influenza virus-specific antibody in ferrets, we then assessed whether it was protective against infectious challenge.

### saRNA vaccine accelerates influenza virus clearance in ferrets

**Figure 3:**
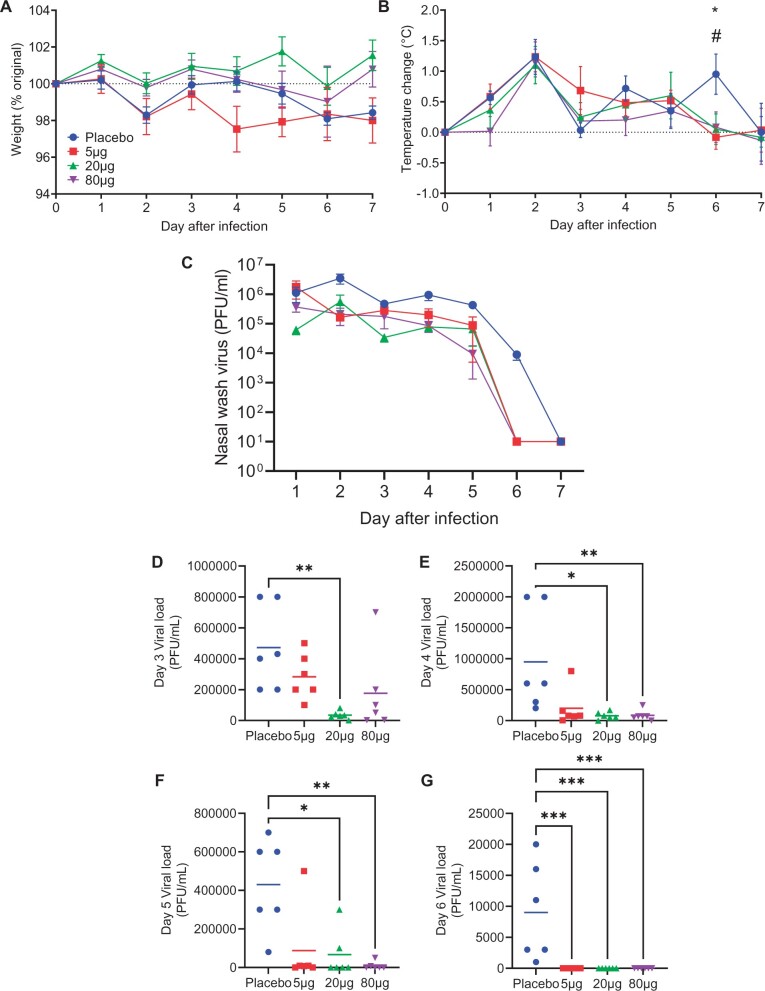
pABOL-formulated saRNA provides some protection against influenza infection. Female ferrets were intramuscularly immunized with 5, 20 or 80 µg saRNA encoding HA formulated with pABOL at 0 and 4 weeks. They were then challenged intranasally with influenza at 6 weeks. Weight (**A**) and temperature (**B**) change after infection. Viral load timecourse (**C**) and daily loads (**D**–**G**). *N* = 6 ferrets per group, points represent mean ± SEM (A–C) individual animals (D–G); Kruskal Wallis test **P < 0.01, ***P < 0.001.

Immunized ferrets were challenged intranasally with influenza virus at 6 weeks. There was no difference between the groups in weight loss after infection (Fig. 3A). The animals were also monitored for temperature changes. There was a spike in temperature at d2 in all animals and the placebo group had a significantly elevated temperature at d6 after infection (Fig. 3B). Viral load was assessed over the time of experiment—presented as timecourse (Fig. 3C) and a day by day breakdown (Fig. 3D–G). The 20-µg group had significantly less virus than the placebo from days 3 to 7 and the 80-µg group had less virus from days 4 to 7. Vaccinated animals cleared virus a day sooner than control animals.

**Figure 4:**
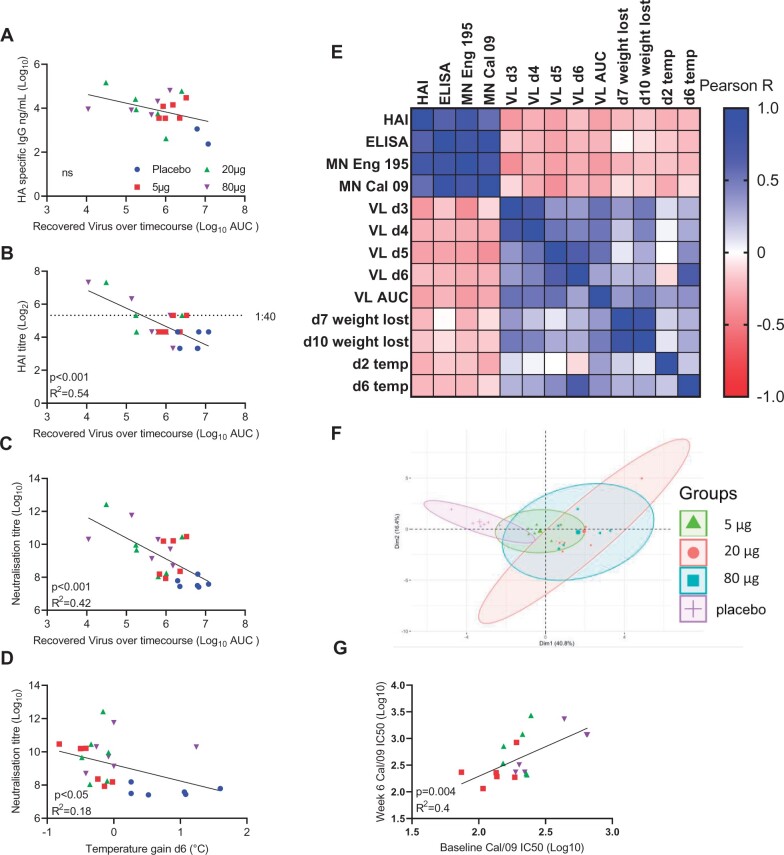
Vaccine-induced antibodies correlate with protection. Comparison of antibody and viral load, ELISA (**A**), HAI (**B**), microneutralization (**C**), comparison of neutralizing antibody and d6 temperature (**D**). Correlation of antibody, viral load and disease characteristics (**E**). PCA of multiple datapoints (**F**). Correlation of baseline and week 6 Cal/09 microneutralization titre (**G**). *N* = 6 ferrets per group, points represent individual animals.

Having observed a variable antibody response after vaccination and variable levels of protection against infection, we investigated whether there was a link between the response and protection. We performed a *post hoc* analysis of the correlation between antibody and protection against infection. We used area under the curve (AUC) of the virus recovered over the timecourse of the study as an estimate of the total viral load. There was no significant correlation between binding ELISA titre and viral clearance (Fig. 4A). However, there was a significant inverse correlation between HAI titre (*R*^2^ = 0.54, Fig. 4B), neutralization titre (*R*^2^ = 0.42, Fig. 4C) and virus recovered; indicating that when antibody was induced by the vaccine, it reduced the viral load. There was also a weakly significant correlation between neutralizing titre and temperature gain at day 6 (Fig. 4D). We performed a more global analysis of antibody, viral load and clinical signs (Fig. 4E). Antibody titres—binding and functional closely correlated as did viral load and temperature/weight loss; there was an inverse correlation between antibody and viral load and clinical signs of disease. Performing a PCA on the clinical, viral and immunological data demonstrated a separation between the placebo and immunized groups (Fig. 4F), suggesting there is an impact of immunization, but that the degree of protection can be variable. We compared whether there was any association between baseline and week 6 antibody response, in case prior asymptomatic exposure had an impact, there was no association for HAI or ELISA, but there was a significant correlation between baseline and week 6 neutralization titre for Cal/09 (Fig. 4G).

### Protection against H3N2 transmission

**Figure 5:**
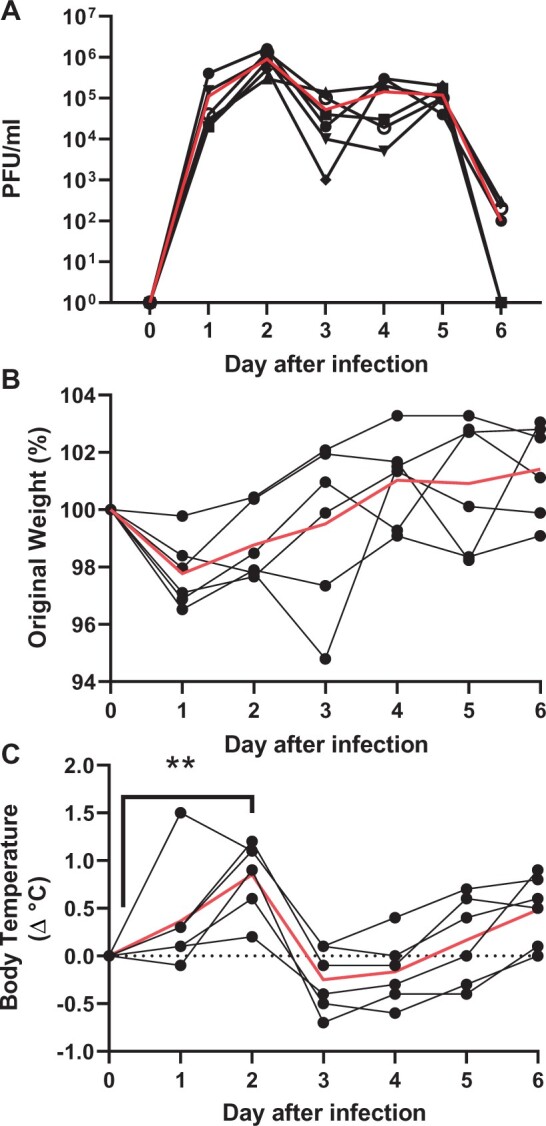
Establishing H3N2 infection in ferrets. Adult ferrets were infected intranasally with 5 × 10^5^ PFU H3N2 Japan (A_JapanWRAIR1059P_2009). These animals were used as donor ferrets for the transmission study. Nasal samples were collected daily after infection and assessed for viral load (**A**). Weight (**B**) and temperature (**C**) change after infection. *N* = 6. Red line represents mean. **P < 0.01 between day 0 and d2, by Kruskal–Wallis and post test.

Having observed that the pABOL-formulated saRNA gave some, but variable, protection against H1N1 influenza virus infection, we wished to investigate whether a saRNA vaccine could be protective against a broader range of influenza virus infections. To test this, we established a model of H3N2 infection. Adult ferrets were intranasally infected with 5 × 10^5^ H3N2 Japan (A/Japan/WRAIR1059P/2008(H3N2)). Nasal wash samples were collected daily to assess viral shedding, there was detectable virus from day 1 to day 6, and most animals still had virus on day 6 after infection (Fig. 5A). There was no detectable weight loss after infection (Fig. 5B), but there was a spike in temperature on day 2 after infection that was significantly higher than baseline (Fig. 5C). These animals were used as donor animals for the protection against transmission study.

**Figure 6:**
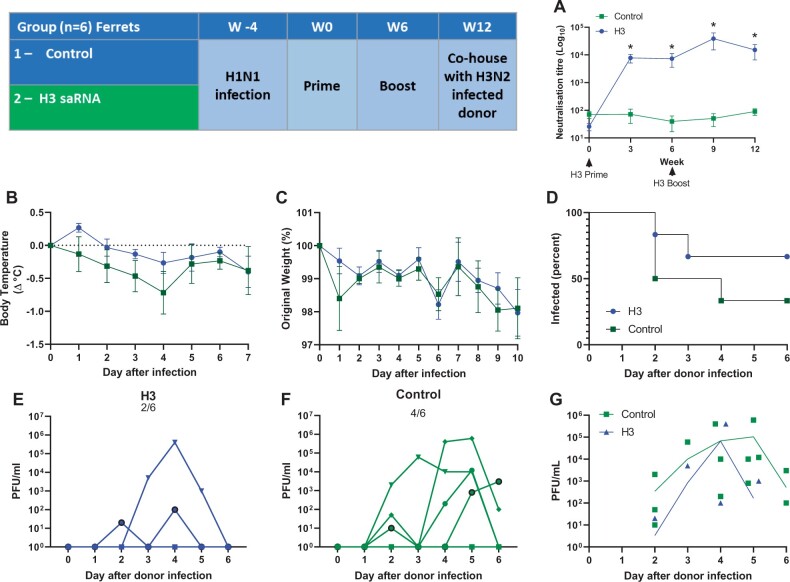
Testing an H3N2 saRNA vaccine for protection against in cage transmission. Female ferrets were immunized with pABOL-formulated saRNA expressing H3N2 or RABV (as a control antigen) in a 0- and 6-week prime-boost regime according to schedule. H3N2 neutralizing antibody titre (**A**). At week 12, immunized animals were housed with a donor animal that had been infected 1 day previously (three animals to a cage: one donor, one H3 and one control). Temperature change (**B**) and weight (**C**) were measured after infection. Number of animals that were viral positive (**D**), Nasal wash viral load recorded for individual animals (**E** and **F**) and both groups (**G**).

Because influenza virus is endemic, we wished to mimic previous viral exposure to a different strain. Four weeks prior to the first immunization, the immunized animals in the study were initially infected with 10^6^ PFU H1N1. Following this infection, adult female ferrets were immunized in a prime-boost regime with 20 µg saRNA in the hindleg at 0 and 6 weeks. Influenza virus-specific antibody was assessed by pseudo-neutralization assay (Fig. 6A). The H3 immunized animals produced influenza virus neutralizing antibody, this was significantly greater than the control animals that were only immunized with control saRNA expressing rabies virus glycoprotein. To test for protection against influenza virus infection, we used a contact transmission model [20]. Previously naïve, H3N2-infected donor animals (characterized in Fig. 5) were housed with the immunized or control animals, one donor animal per cage. The animals were co-housed 1 day after infection of the donor animals. Infection was then assessed through viral load and signs of disease. There was no difference in temperature change (Fig. 6B) or weight loss (Fig. 6C) between the H3 immunized and control groups. Viral load was assessed by plaque assay, two out of six H3 immunized ferrets were infected compared with four out of six control animals (Fig. 6D–F). There was no difference in the mean viral load in the infected animals (Fig. 6G).

## DISCUSSION

In the current study, we investigated the immunogenicity of a polymer-formulated saRNA vaccine against influenza virus in ferrets. We observed variable responses between different immunized animals, those that did produce a strong neutralizing antibody response were better protected against influenza virus challenge. Previous studies have looked at RNA vaccination against influenza virus in ferrets. The majority of these looked at non-replicating mRNA vaccines, and observed variable antibody responses to the prime immunization, with some animals not responding [10, 11]. Interestingly, responses were variable even after the incorporation of modified nucleosides, which have worked well in the COVID vaccines [12]. Reflecting the relative novelty of the platform, we could only find one previously published study in ferrets using saRNA. In that study which used an LNP-formulated saRNA vaccine, the authors reported a similar variable response to the vaccine, with lower viral load at d5 after infection, but not all animals cleared the infection [13]. The rapid progress in RNA vaccines since 2020 has led to new phase I clinical trials of RNA vaccines against influenza virus from both Moderna (NCT04956575) and Pfizer/BioNTech (NCT05052697) among others. The use of ferret models in parallel with these studies may help to identify correlates of immunogenicity and protection against infection or disease.

The variability of response was in contrast to mice which respond with less variability to low doses of saRNA formulated in pABOL in this study and previous studies [6, 16]. The variability observed in the ferrets is similar to that seen in a recently performed first in human phase I trial of a saRNA SARS-CoV-2 vaccine [4]. Why there is variability in ferrets, and whether the reason for it reflects the human response, is currently unclear. One possible factor is how the RNA in the vaccine is sensed; unlike other platforms, RNA vaccines need to be translated prior to the induction of an immune response [27]. It was of note, that there was a dose response, with a prozone effect around the middle dose, suggesting the vaccine material itself may be inhibitory at higher doses, this has been observed in other studies [28]; though this could be an effect of formulation. There are differences between mice and humans in the sensing for RNA, for example TLR8 in inbred mice has a different expression pattern and sensitivity to ligands than human TLR8 [29], lacking five amino acids in the ectodomain that may contribute to functionality [30]. Whether the sensing of RNA vaccines by ferrets more closely mimics mice or humans is not yet well established. The ferret TLR8 gene does encode amino acids (similar to the cat) at the PGIQ and RQYS motifs that are missing from the mouse TLR8 (comparing human TLR8 NP_057694.2 and ferret XP_044945234.1) and some sensitivity to TLR8 ligands has been reported for isolated ferret cells [31]. A recent study has observed immunogenicity of saRNA vaccines in golden hamsters [32], which are also lacking the PGIQ motif (XP_021085220.1) in TLR8. Another difference may relate to the previous exposure to the environment. The mice used in the immunization studies are gnotobiotic, raised in relatively pathogen-free conditions, whereas ferrets are reared in larger barns prior to studies. This natural history may affect sensitivity to other agents and may more closely reflect human vaccine trial volunteers who may be sub-clinically infected with pathogens at the time of immunization. Prior exposure may also affect the microbiome, with laboratory mice having less microbiome diversity and ‘wild’ mice responding differently to vaccination [33, 34]. In addition to impact on sensing of the formulated vaccine, there may need to be species-specific optimization of RNA vaccines as there may be differences in translation machinery between the species.

In the ferrets that did respond to the H1 vaccine, neutralizing antibody significantly correlated with accelerated clearance of influenza virus and a reduced overall viral load. It was notable that binding antibody measured by ELISA didn’t correlate with reduced viral load, suggesting that to reduce infection the antibody needs to be raised against regions of the virally expressed HA protein required for cell entry. The H1N1 infection model was relatively mild, with only a modest weight loss in the placebo-vaccinated group, however there was a spike in temperatures in all animals on day 2 after infection and second spike on d6 after infection. While neutralizing antibody titre did not correlate with d2 temperature, there was a weakly significant association with d6 temperature, suggesting that induced antibody was partially protective against signs of disease. It was interesting to note that none of the vaccines gave complete, sterilizing immunity with no recoverable virus. This is not uncommon in ferret studies—for example there was detectable virus after immunization with an HA stem fragment [35] (which differed to the mouse model in the same study), a live attenuated virus vaccine [36] or an adjuvanted protein vaccine [37]. Direct neutralization may not be the only function of anti-HA antibodies in protection, we have previously observed that NK cells are required for vaccine protection [38] and a recent study showed an association between NK activating antibodies and lower respiratory tract control of RSV [39]. These functional, but non-neutralizing antibodies may bind other, more conserved regions of HA, for example the stem. A recent study demonstrated heterotypic protection in ferrets immunized with a nanoparticle engineered to induce anti-stem antibodies [40]. A different approach to thinking about what defines protection for a range of vaccines may be required, for example SARS-CoV-2 infection as measured by RNA has been observed in vaccinated individuals [41] as has onwards transmission, but there has been a significant reduction in disease [42]. One aspect that we did not explore in this study is the role of mucosal antibody [43], intramuscular immunization does not induce as strong mucosal responses which may explain the limited protection against infection offered by this vaccine. Going forward, better induction of local antibody may be beneficial for anti-viral vaccines.

While there was some reduction in viral load in the vaccinated animals, it was not optimal and more work is required to improve the immunogenicity of saRNA vaccines, this reflects the clinical trial experience with an equivalent construct [4], although with a LNP formulation. LNP may induce a different immune profile to vaccination in ferrets, as was seen in mice [16]. Dampening the type I interferon response is one potential approach to improve saRNA vaccine immunogenicity and has been extremely effective in the mRNA vaccines for COVID-19, through RNA silencing [44]. An alternative is to dampen the host response, we have previously seen in mice that inclusion of MERS-CoV ORF4A or PIV-5 V proteins, both of which target MDA5, can increase expression [28]. Other studies have suggested the incorporation of steroid precursors into saRNA vaccine formulation as an approach to dampen the immune response [45]. It is possible that formulation of higher concentrations of RNA altered the transfection efficiency of the polyplex [6].

There are some limitations of the studies presented here. Previous, asymptomatic exposure to a related influenza virus may also contribute to some variability in the response, as animals with a higher baseline neutralization titre had higher week six titres. However, previous exposure was not enough to protect against further infection, as the placebo animals were unprotected and previous infection was insufficient to lead to an NP antibody response, though there was some baseline HAI. Both the seasonal influenza virus infections, H1N1 and H3N2, were relatively mild in the ferrets, so it was difficult to investigate the impact on disease rather than viral shedding.

In the current study, we investigated formulation of saRNA with pABOL, a bioreducible polymer as a way to immunize ferrets against influenza virus. We saw variable degrees of antibody induction after vaccination associated with variable protection against viral infection. Understanding more about saRNA vaccine sensing and the impact of basal immune ‘tone’ will be important in the further development of this platform. More research about the response to RNA vaccines in the ferret model is needed; particularly, in understanding how similar the responses are to humans with regards to RNA sensing and downstream pathways that may influence expression.

## DATA AVAILABILITY STATEMENT

Raw data can be obtained from corresponding authors on request.

## References

[1] Tregoning JS , Brown ES, Cheeseman HM et al Vaccines for COVID-19. Clin Exp Immunol 2020;202:162–92. 32935331 10.1111/cei.13517 PMC7597597

[2] Blakney AK , Ip S, Geall AJ. An update on self-amplifying mrna vaccine development. Vaccines (Basel*)* 2021;9. 10.3390/vaccines9020097 PMC7911542 33525396

[3] Vogel AB , Lambert L, Kinnear E et al Self-amplifying RNA vaccines give equivalent protection against influenza to mRNA vaccines but at much lower doses. Mol Ther 2018;26:446–55. 29275847 10.1016/j.ymthe.2017.11.017 PMC5835025

[4] Pollock KM , Cheeseman HM, Shattock RJ. Safety and Immunogenicity of a Self-Amplifying RNA Vaccine Against COVID-19: COVAC1, a Phase I, Dose-Ranging Trial. Preprint, 2021. 10.1016/j.eclinm.2021.101262 PMC8759012 35043093

[5] Maruggi G , Ulmer JB, Rappuoli R et al Self-amplifying mRNA-Based Vaccine Technology and Its Mode of Action. Berlin: Springer. p. 1–40. 10.1007/82_2021_233 33861374

[6] Blakney AK , Zhu Y, McKay PF et al Big is beautiful: Enhanced saRNA delivery and immunogenicity by a higher molecular weight, bioreducible, cationic polymer. ACS Nano 2020;14:5711–27. 32267667 10.1021/acsnano.0c00326 PMC7304921

[7] Moore KA , Ostrowsky JT, Kraigsley AM et al A Research and Development (R&D) roadmap for influenza vaccines: Looking toward the future. Vaccine 2021;39:6573–84. 34602302 10.1016/j.vaccine.2021.08.010

[8] Paget J , Spreeuwenberg P, Charu V et al Global mortality associated with seasonal influenza epidemics: New burden estimates and predictors from the GLaMOR project. J Glob Health 2019;9:020421. 31673337 10.7189/jogh.09.020421 PMC6815659

[9] Belser JA , Barclay W, Barr I et al Ferrets as models for influenza virus transmission studies and pandemic risk assessments. Emerg Infect Dis 2018;24:965–71. 29774862 10.3201/eid2406.172114 PMC6004870

[10] Bahl K , Senn JJ, Yuzhakov O et al Preclinical and clinical demonstration of immunogenicity by mRNA vaccines against H10N8 and H7N9 influenza viruses. Mol Therapy 2017;25:1316–27. 10.1016/j.ymthe.2017.03.035 PMC5475249 28457665

[11] Petsch B , Schnee M, Vogel AB et al Protective efficacy of in vitro synthesized, specific mRNA vaccines against influenza A virus infection. Nat Biotechnol 2012;30:1210–6. 23159882 10.1038/nbt.2436

[12] Pardi N , Parkhouse K, Kirkpatrick E et al Nucleoside-modified mRNA immunization elicits influenza virus hemagglutinin stalk-specific antibodies. Nat Commun 2018;9:3361. 30135514 10.1038/s41467-018-05482-0 PMC6105651

[13] Brazzoli M , Magini D, Bonci A et al Induction of broad-based immunity and protective efficacy by self-amplifying mRNA vaccines encoding influenza virus hemagglutinin. J Virol 2016;90:332–44. 26468547 10.1128/JVI.01786-15 PMC4702536

[14] Hoerr I , Obst R, Rammensee H-G et al In vivo application of RNA leads to induction of specific cytotoxic T lymphocytes and antibodies. Eur J Immunol 2000;30:1–7. 10602021 10.1002/1521-4141(200001)30:1<1::AID-IMMU1>3.0.CO;2-#

[15] McKay PF , Hu K, Blakney AK et al Self-amplifying RNA SARS-CoV-2 lipid nanoparticle vaccine candidate induces high neutralizing antibody titers in mice. Nat Commun 2020;11:3523. 32647131 10.1038/s41467-020-17409-9 PMC7347890

[16] Blakney AK , McKay PF, Hu K et al Polymeric and lipid nanoparticles for delivery of self-amplifying RNA vaccines. J Control Release 2021;338:201–10. 34418521 10.1016/j.jconrel.2021.08.029 PMC8412240

[17] Russell RF , McDonald JU, Lambert L et al Use of the microparticle NanoSiO_2_ as an adjuvant to boost vaccine immune responses in neonatal mice against influenza. J Virol 2016;90:4735–44. 26912628 10.1128/JVI.03159-15 PMC4836350

[18] Lambert L , Kinnear E, McDonald JU et al DNA vaccines encoding antigen targeted to MHC class II induce influenza-specific CD8+ T cell responses, enabling faster resolution of influenza disease. Front Immunol 2016;7. 10.3389/fimmu.2016.00321 PMC4993793 27602032

[19] Lee LYY , Zhou J, Frise R et al Baloxavir treatment of ferrets infected with influenza A(H1N1)pdm09 virus reduces onward transmission. PLoS Pathog 2020;16:e1008395. 32294137 10.1371/journal.ppat.1008395 PMC7159184

[20] Roberts KL , Shelton H, Stilwell P et al Transmission of a 2009 H1N1 pandemic influenza virus occurs before fever is detected, in the ferret model. PLoS ONE 2012;7:e43303. 22952661 10.1371/journal.pone.0043303 PMC3430703

[21] R Core Team. *R: A language and environment for statistical computing*. R Foundation for Statistical Computing, Vienna, Austria. 2018. Available online at https://www.R-project.org/.

[22] Tang Y , Horikoshi M, Li W. ggfortify: unified interface to visualize statistical result of popular R packages. R Journal 2016;8:2.

[23] Ligges U , Maechler M. Scatterplot3d—an R package for visualizing multivariate data. J Statist Softw 2003;8:1–20.

[24] Sheppard NC , Brinckmann SA, Gartlan KH et al Polyethyleneimine is a potent systemic adjuvant for glycoprotein antigens. Int Immunol 2014;26:531–8. 24844701 10.1093/intimm/dxu055

[25] Groves HT , McDonald JU, Langat P et al Mouse models of influenza infection with circulating strains to test seasonal vaccine efficacy. Front Immunol 2018;9. 10.3389/fimmu.2018.00126 PMC5797846 29445377

[26] Hobson D , Curry RL, Beare AS et al The role of serum haemagglutination-inhibiting antibody in protection against challenge infection with influenza A2 and B viruses. J Hyg 1972;70:767–777. 4509641 10.1017/s0022172400022610 PMC2130285

[27] Minnaert A-K , Vanluchene H, Verbeke R et al Strategies for controlling the innate immune activity of conventional and self-amplifying mRNA therapeutics: Getting the message across. Adv Drug Deliv Rev 2021;176:113900. 34324884 10.1016/j.addr.2021.113900 PMC8325057

[28] Blakney AK , McKay PF, Bouton CR et al Innate inhibiting proteins enhance expression and immunogenicity of self-amplifying RNA. Mol Ther 2021;29:1174–85. 33352107 10.1016/j.ymthe.2020.11.011 PMC7935664

[29] Barrat FJ. TLR8: No gain, no pain. J Exp Med 2018;215:2964–6. 30455266 10.1084/jem.20181899 PMC6279395

[30] Liu J , Xu C, Hsu L-C et al A five-amino-acid motif in the undefined region of the TLR8 ectodomain is required for species-specific ligand recognition. Mol Immunol 2010;47:1083–90. 20004021 10.1016/j.molimm.2009.11.003 PMC2815190

[31] Berendam SJ , Fallert Junecko BA, Murphey-Corb MA, et al Isolation, characterization, and functional analysis of ferret lymphatic endothelial cells. Vet Immunol Immunopathol 2015;163:134–45. 25540877 10.1016/j.vetimm.2014.11.013 PMC4323863

[32] Frise R , Baillon L, Zhou J et al A self-amplifying RNA vaccine protects against SARS-CoV-2 (D614G) and Alpha variant of concern (B.1.1.7) in a transmission-challenge hamster model. Vaccine 2022;40:2848–55. 35396165 10.1016/j.vaccine.2022.03.064 PMC8971064

[33] Rosshart SP , Herz J, Vassallo BG et al *.* Laboratory mice born to wild mice have natural microbiota and model human immune responses. Science 2019;365:eaaw4361. 31371577 10.1126/science.aaw4361 PMC7377314

[34] Fiege JK , Block KE, Pierson MJ et al Mice with diverse microbial exposure histories as a model for preclinical vaccine testing. Cell Host Microbe 2021;29(12). 10.1016/j.chom.2021.10.001 PMC8665115 34731647

[35] Sutton TC , Chakraborty S, Mallajosyula VVA et al Protective efficacy of influenza group 2 hemagglutinin stem-fragment immunogen vaccines. NPJ Vaccines 2017;2:35. 29263889 10.1038/s41541-017-0036-2 PMC5732283

[36] Czakó R , Vogel L, Sutton T et al H5N2 vaccine viruses on Russian and US live attenuated influenza virus backbones demonstrate similar infectivity, immunogenicity and protection in ferrets. Vaccine 2018;36:1871–9. 29503113 10.1016/j.vaccine.2018.02.061 PMC5854182

[37] Christensen D , Christensen JP, Korsholm KS et al Seasonal influenza split vaccines confer partial cross-protection against heterologous influenza virus in ferrets when combined with the CAF01 adjuvant. Front Immunol 2017;8:1928. 29358939 10.3389/fimmu.2017.01928 PMC5766649

[38] Mooney JP , Qendro T, Keith M et al Natural killer cells dampen the pathogenic features of recall responses to influenza infection. Front Immunol 2020;11:135. 32117282 10.3389/fimmu.2020.00135 PMC7019041

[39] Zohar T , Hsiao JC, Mehta N et al Upper and lower respiratory tract correlates of protection against respiratory syncytial virus following vaccination of nonhuman primates. Cell Host Microbe 2022;30:41–52.e5. 34879230 10.1016/j.chom.2021.11.006

[40] Boyoglu-Barnum S , Ellis D, Gillespie RA et al Quadrivalent influenza nanoparticle vaccines induce broad protection. Nature 2021;592:623–8. 33762730 10.1038/s41586-021-03365-x PMC8269962

[41] Singanayagam A , Hakki S, Dunning J et al *. Community Transmission and Viral Load Kinetics of the SARS-CoV-2 Delta (B.1.617.2) Variant in Vaccinated and Unvaccinated Individuals in the UK: A Prospective, Longitudinal, Cohort Study*. The Lancet Infectious Diseases. 10.1016/S1473-3099(21)00648-4 PMC8554486 34756186

[42] Tregoning JS , Flight KE, Higham SL et al Progress of the COVID-19 vaccine effort: viruses, vaccines and variants versus efficacy, effectiveness and escape. Nat Rev Immunol 2021;21:626–636. 34373623 10.1038/s41577-021-00592-1 PMC8351583

[43] Lavelle EC , Ward RW. Mucosal vaccines—fortifying the frontiers. Nat Rev Immunol 2022;22:236–50. 34312520 10.1038/s41577-021-00583-2 PMC8312369

[44] Chaudhary N , Weissman D, Whitehead KA. mRNA vaccines for infectious diseases: principles, delivery and clinical translation. Nat Rev Drug Discov 2021;20:817–38. 34433919 10.1038/s41573-021-00283-5 PMC8386155

[45] Davies N , Hovdal D, Edmunds N et al Functionalized lipid nanoparticles for subcutaneous administration of mRNA to achieve systemic exposures of a therapeutic protein. Mol Therapy 2021;24:369–84. 10.1016/j.omtn.2021.03.008 PMC8039535 33868782

